# Late registration for antenatal care by pregnant women with previous history of caesarean section

**DOI:** 10.4102/phcfm.v13i1.2776

**Published:** 2021-05-26

**Authors:** Mareko Ramotsababa, Vincent Setlhare

**Affiliations:** 1 Ministry of Health and Wellness, Government of Botswana, Gaborone, Botswana; 2 Department of Family Medicine and Public Health Medicine, Faculty of Medicine, University of Botswana, Gaborone, Botswana

**Keywords:** previous caesarean section, maternal risk, late registration for antenatal care, late presentation for antenatal care

## Abstract

**Background:**

Despite good access to antenatal care (ANC) services for most women, and regular training of healthcare workers in obstetrics and gynaecology, many pregnant women with a previous history of caesarean section (C/S) still presented late for ANC services at Letsholathebe II Memorial Hospital (LIIMH) in Maun, Botswana. This may increase morbidity and mortality in women with previous C/S delivery and neonates. Knowing why women with previous C/S present late for ANC may help in the formulation of interventions that decrease morbidity and mortality amongst these women and neonates.

**Aim:**

The aim of this study was to explore the reasons why pregnant women with a previous history of C/S registered late for ANC, at LIIMH.

**Setting:**

This study was performed at LIIMH, a district hospital situated in Maun, Botswana.

**Methods:**

This was a descriptive qualitative study. Consenting pregnant women with previous C/S who presented at LIIMH after the 20 weeks of pregnancy were interviewed until data saturation. The data was analysed for themes.

**Results:**

The reasons for late registration at LIIMH include lack of information, misconception on the appropriate booking time and venue, dissatisfaction with the quality of ANC clinic services, use of alternative ANC providers, delayed referral, and pregnancy experience.

**Conclusion:**

Lack of knowledge of ANC delivery venue, using alternative ANC providers, and dissatisfaction with ANC clinic services, contributed to late registration. Pregnant women with previous history of C/S should be informed about ANC, delivery plans, and the assistance of alternative ANC providers should be explored.

## Introduction

In Botswana, studies report increased morbidity and mortality rates in patients with or without a previous history of caesarean section (C/S) delivery, who present late in pregnancy.^1,2^ That is why pregnant women should be advised to register for antenatal care (ANC) services in the first trimester and to continue availing themselves for that service throughout the pregnancy.^3^ It is also appropriate that women should be assisted to plan for their next pregnancy, especially if they have conditions that may affect maternal or foetal pregnancy outcomes.^4^ As healthcare services are accessible to most patients in Botswana, it is important to know why some patients with previous history of C/S still present at healthcare facilities late in pregnancy, or in labour, thus exposing themselves to an increased risk of morbidity and mortality. Knowledge of reasons for late registration for ANC may help in the formulation of interventions to reduce morbidity and mortality rates in pregnant women with previous C/S deliveries. To our knowledge, no studies had been performed at Letsholathebe II Memorial Hospital (LIIMH) to explore the reasons why pregnant women with a previous history of C/S registered late for ANC.

The global maternal mortality rate (MMR) in 2017 was estimated to be 211 per 100 000 live births, while the MMR for Botswana in the same year was estimated to be 144.^5^ The highest MMR (415) was reported in the least developed countries, while developed countries the MMR was in single digits.^5^

It is relatively safer to be pregnant and deliver in the United Kingdom (UK) than in Botswana. The series of problems associated with pregnancy and delivery in the UK is different from those leading to MMR in least developed countries.^4,5,6,7^ The low MMR in the UK is because of many factors, such as counselling pregnant women, referring them to appropriate care; implementation of guidelines, and improved policies, procedures and clinical care.^4,5,7^

In Botswana, Mogobe et al. found that out of 36 304 deliveries, 7073 (about 20%) women experienced complications of childbirth.^8^ The study by Mogobe et al. of 14 hospitals in Botswana revealed that in 2005, out of 32 maternal deaths, postpartum haemorrhage accounted for 34% of deaths, obstructed labour 25%, ruptured uterus 20%, eclampsia 16%, antepartum haemorrhage 3% and abortion 3%. The 2018 United Nations Population Fund report for Botswana estimated the maternal mortality ratio in 2015 to be around 129 deaths per 100 000 live births. The maternal mortality ratio in Botswana was estimated at 182.6, 151.6, 127, 156.6 and 143.2 in years 2013, 2014, 2015, 2016 and 2017, respectively.^9^ The maternal mortality ratios in Botswana are very high when compared with the single-digit figures obtained in the UK.^4,7^ In the years 2012–2015, 34% of maternal deaths reported in Botswana were of women who either delayed initiating ANC or never attended ANC.^10^ The slow decline of maternal deaths in Botswana may be attributed to substandard care and deficiencies in the health system, such as equipment and drug shortages, barriers to accessing emergency healthcare services, a dysfunctional referral system and disjointed use of available expertise.^11,12^

For pregnancy outcomes to be improved, there should be adequate pre-pregnancy care, routine ANC visits should begin as early as the first trimester (up to 12 weeks), and ANC visits should continue in the second and third trimesters for a total of four ANC visits, which were later revised to eight visits.^3,12,13,14^ Although early initiation of ANC improved across the world in the period 1990–2013, this did not improve as much in developing countries.^12^ A Nigerian study showed that the initial booking for ANC ranged from 6 weeks to 39 weeks gestation, with a mean of 21.09 weeks.^15^ In Myanmar, the initial booking for ANC was after the first trimester, with 56% of pregnant women registering for the first time at 16 weeks gestation; with a range of 7–34 weeks.^16^

The World Health Organization (WHO) has shown that in Africa over 69% of women have at least one ANC contact during a pregnancy, but four ANC contacts were at a much lower percentage.^13^ Inadequate use of ANC is compounded by late initiation of ANC visits in developing countries.^17^ It was estimated that only 43% of women received ANC before the end of the first trimester in Durban, South Africa,^18^ while in Ethiopia, only 21.72% of pregnant women had their first ANC booking in the first trimester.^19^ Suboptimal ANC utilisation is one of the causes of poor maternal pregnancy outcomes.^20^

The WHO recommends that all pregnant women should have a written plan for delivery, for dealing with complications of delivery and for management of problems of the immediate postnatal period.^14^ In Botswana, the delivery plan is normally documented in an antenatal card that is freely available at government healthcare facilities. It is beneficial for the patients to discuss and review these plans with skilled attendants at every ANC visit and at least a month before the expected date of delivery.^14^ Appropriate use of ANC by pregnant patients facilitates good outcomes of pregnancy for both the mother and child.^4,21^ In Botswana, less than half of the pregnant women began ANC attendance in the first trimester, most were attended to by midwives and nurses, and two-thirds of those in the age group 25–34 years received adequate ANC visits, while ANC visits were lowest amongst the age group 15–24 years.^22^ The proportion of pregnant women in Botswana who had four or more ANC visits was 73.3% during the period 2005–2007.^23^

The findings of a 2011 study in the UK revealed that both patient and healthcare system are the key factors that delayed access to ANC.^24^ Other factors that compromised proper use of ANC included delayed diagnosis, poor reproductive health knowledge, contraceptive failure, lack of assistance in engagement with ANC and experience from previous pregnancies.^24^

In sub-Saharan Africa, higher education of a pregnant woman and her husband with, being employed, being married, increasing age, higher socio-economic status and involvement in decision-making on important subjects were positively associated with the extent of ANC utilisation and, in some instances, early initiation thereof.^4,25^ Increasing parity, rural residence, low socio-economic status and long distance from a healthcare facility were negatively associated with the extent of use and early initiation of ANC.^14,25^ In the Niger Delta, Nigeria, most women delayed in receiving ANC service because they believed that booking in the first three months of pregnancy confers no advantages, and they also thought that ANC was more of a curative service than preventive.^26^ Women also presented late because of their need for secrecy in early pregnancy, preference for traditional midwives and presumption of multiparous women that they were experienced in pregnancy.^27^ In Addis Ababa, Ethiopia, late initiation of ANC amongst pregnant women was associated with low educational level, low income status, unplanned pregnancy, lack of information about ANC and cost of ANC.^28^ In Nepal, late booking for ANC by pregnant women was associated with low education level, lower socio-economic status, religious background and region.^29^

Antenatal care should include categorising patients according to levels of risk to determine the level of healthcare professional to care for the patient, and the type of healthcare facility to provide the care.^3,4^ This helps to improve management of problems of pregnancy, delivery and the post-delivery period.

Patients with a previous history of C/S (even one) delivery, who attempt vaginal birth, are at risk of undergoing emergency C/S, rapture of uterus and losing their baby.^30,31^ In Scotland women who had a planned vaginal birth after C/S were significantly more likely to have uterine rupture, blood transfusion, surgical injury, puerperal sepsis, perinatal deaths and perinatal admissions, compared with those who delivered by an elective C/S after a previous C/S delivery.^32^ When vaginal birth after previous C/S was attempted, 28% – 55% of women ended up having an emergency C/S during labour.^32,33^ In low-to-middle-income countries (LMICs), women undergoing an emergency C/S are twice as likely to die than those undergoing an elective C/S, and the risk increases 12 times if the C/S is performed in the later stages of labour.^34^ In Thailand, factors influencing post- C/S morbidity included having less than four ANC visits and duration of labour longer than 12 h.^16^ However, evidence suggests that vaginal birth can be safely achieved in women with previous C/S delivery.^31^

In Scotland, 28% of 28 464 women who attempted vaginal delivery after a previous C/S delivery had an emergency C/S during labour.^32^ Emergency C/S is associated with more fresh stillbirths, neonatal deaths, severe neonatal morbidity and increased maternal mortality compared with elective C/S delivery.^34,35^ Providing improved care after C/S may decrease MMRs caused by treatable conditions, such as post-partum haemorrhage, post-operative fever, wound infection, anaesthesia-related complications and pre-eclampsia.^34,35^

The Royal College of Obstetricians and Gynaecologist (UK) recommends that pregnant women with a previous history of C/S delivery should consult healthcare professionals from 12 weeks onwards for discussions on their next mode of delivery and other ANC-related issues.^36^

The WHO millennium development goal number five (MDG-5) aimed at achieving a 75% reduction in MMRs by 2015. As a result, the Ministry of Health (Botswana), in March 2011, launched the Campaign for Accelerated Reduction of Maternal Mortality in Africa (CARMMA) in Maun. The Ministry also started a 2-week course for healthcare professionals called Emergency Management of Obstetric and Neonatal Cases (EMONC) aimed at helping to reduce maternal and neonatal mortality. As a result, doctors in LIIMH decided that all patients with previous history of C/S delivery should not undergo vaginal delivery. This approach is in line with one of the studies in Scotland, which showed that planned vaginal delivery carries far more risk in women with previous C/S than repeat elective C/S.^32^ Doctors at LIIMH suggested that women with previous history of C/S required full ANC and an appropriate plan for managing their delivery during their current pregnancy.

Despite education about the importance of appropriate use of ANC, pregnant patients, including those with a previous history of C/S, still presented late for ANC or presented in labour at LIIMH. We thought that it was important to explore why patients presented late for ANC, thus putting their lives and those of their babies at risk.

## Methods

The aim of this study was to explore the reasons for late presentation for ANC at LIIMH in Maun, Botswana, by adult pregnant women with a previous history of C/S delivery after 20 weeks gestation and to suggest preliminary recommendations that would encourage these women to present themselves for ANC before 20 weeks gestation.

For the purposes of this study, women who presented before 20 weeks gestation in lower-level healthcare facilities, but were referred to LIIMH after 20 weeks gestation, were considered to have presented themselves late.

This was a descriptive qualitative study, in which structured interviews were used to collect data. The study site was LIIMH, a referral district hospital situated in Maun, Botswana. Maun is the gateway to the Okavango delta and is situated in a district where people are engaged in tourism, agriculture and working for the government in various sectors. Letsholathebe II Memorial Hospital is a 300-bed referral hospital for three primary hospitals, 10 primary care clinics, and many health posts. Letsholathebe II Memorial Hospital served a population of 195 371 in 2016, with the hospital recording 2778 births in 2015.^37^ This referral hospital has specialists in obstetrics and gynaecology, as well as in other disciplines; however, because of staff shortages, most pregnant women were attended by non-specialist doctors and midwives.

The study population included pregnant women with a previous history of C/S who presented after 20 weeks of pregnancy at LIIMH. Purposive sampling was used to select participants from pregnant women with a previous history of C/S delivery who were seen for the first time at LIIMH after 20 weeks gestation. Selected participants varied in age, educational level, employment, number of previous C/S deliveries and gestational age at first presentation at LIIMH. These women were likely to have knowledge of why pregnant women with a previous history of C/S delivery presented after 20 weeks gestation at LIIMH.

Pregnant women without a previous history of C/S delivery, with a previous history of C/S who presented before 20 weeks gestation and with previous C/S but were < 18 years old were excluded. Participants were recruited by the researcher (M.R.) and midwives at the high-risk clinic, in antenatal wards and postnatal wards of LIIMH. M.R. and midwives recruited eligible patients who were given information sheets that explained the study. M.R. and recruiting midwives answered all queries raised by potential participants. They also informed participants that they were not obliged to participate, and that they could decline to participate, or withdraw from the study at will, without any negative repercussions from the hospital or its staff. Participants were also informed by M.R. that the study would be published in a professional journal. Those who were willing to participate and provide audiotaped interviews signed consent forms.

M.R. was a senior resident in a family medicine programme. He received training in both qualitative and quantitative research during his residency. V.S. was M.R.’s supervisor in the residency programme. V.S. had received training in qualitative research, was a trainer in qualitative research in the Family Medicine Residency Programme and had published qualitative research studies. M.R. was not attached to the Obstetrics and Gynaecology Department of the hospital at the time of the study.

M.R. conducted structured interviews using an audio recorder to collect data from participants who had given their consent to be interviewed and audiotaped. He used an interview guide and a questionnaire for demographic information (Appendix Table 1-A1) to collect both demographic data and data relating to why pregnant women with previous history of C/S section presented after 20 weeks gestation at LIIMH for ANC.

Interviews were conducted in Setswana (local language) and were audio recorded. Interview questions were asked in an iterative manner to help in collecting deep and rich data. Field notes were taken to capture data on the interviewer, interviewee and circumstances pertinent to the interview environment. The first two interview recordings were reviewed by M.R. and V.S. V.S. is an experienced qualitative researcher, and he discussed the interviews with M.R. in order to improve the quality of subsequent interviews and to improve the interview guide, so that it was user friendly. Participants were interviewed until data saturation. Data saturation was reached after 10 interviews, when it was deemed that no new information or the type of participants was being added.

The audio interviews were transcribed in Setswana (the local language) by M.R., and then translated into English by M.R. who was a postgraduate student, and very conversant with both English and Setswana. V.S. who is a member of staff in the residency programme in which M.R. was receiving training and is proficient in both English and Setswana compared the Setswana and English versions of three interviews. V.S. was satisfied that the English transcripts reflected the contents of the Setswana transcripts. The data were analysed for themes.^38,39^ M.R. and V.S. independently coded five similar English transcripts after reading them several times. They independently came up with codes, and then agreed on the codes to be used to analyse the data after discussing the codes. M.R. uploaded the English transcripts onto ATLAS-ti© software for analysis using agreed to codes. Any new codes that M.R. found during analysis were discussed with V.S. for consensus.

After analysing the data, codes were merged into themes after discussions between M.R. and V.S. Relationships between themes were discussed between the two, and this further helped to interpret the data.

### Ethical considerations

Ethical approval was obtained from the University of Botswana Institutional Review Board (IRB), Ministry of Health Review Board under licence number – PPME 13/18/1 VIII (467), and permission to conduct the study on site was granted by Ngami DHMT IRB.

## Results

Table 1 shows a summary of the demographic characteristics of 10 participants within the age group 22–35 years.

**TABLE 1:** Participants’ characteristics.

Participant	Sex	Age	Number of previous C/Ss	Gestational age (weeks) when first seen at LIIMH	Education
P1	F	31	1	38	Tertiary
P2	F	30	1	38	Tertiary
P3	F	35	1	37	Secondary
P4	F	30	1	35	Tertiary
P5	F	27	2	40	Secondary
P6	F	22	1	28	Primary
P7	F	27	2	28	Secondary
P8	F	24	1	37	Secondary
P9	F	34	2	38	Primary
P10	F	28	1	32	Secondary

Note: Tertiary: ≥ 12 years of education; secondary: > 7 – < 12 years of education, primary: ≤ 7 years of education.

F, female; C/Ss, caesarean sections; LIIMH, Letsholathebe II Memorial Hospital.

### Reasons for late registration (registration after 20 weeks of pregnancy)

#### Lack of information from healthcare workers

Most participants said they would have presented for ANC at LIIMH earlier if they had known the risks they faced:

‘The main reason is that we don’t know … people don’t know. If I had known that having delivered previously by operation, and that I will need another operation, then I would have long gone to LIIMH knowing that I am in danger so I shouldn’t just be seen at the clinic.’ (P1, aged 31, prev C/S x 1)

#### Misconceptions about antenatal care

Some participants felt that early registration for ANC did not make sense:

‘The thing is if you register after 1 or 2 months, it is still only blood and the baby is not yet formed. I feel that when it’s a properly formed baby, they can massage you and give you pregnancy treatments. The thing is when you are still 1 month; they don’t know what it is inside. It’s just blood.’ (P5, aged 27, prev C/S x 2)

One of the participants believed that the main reason for ANC was the prevention of mother to child transmission (PMTCT) of HIV:

‘[*Y*]ou could even register at 6 or 7 months … because PMTCT program starts when one is at least 6 months …’ (P9, age34, prev C/S x 2)

#### Dissatisfaction with the quality of antenatal care and unmet expectations

Some participants were not happy with the ANC services provided:

‘All she did was take your weight, check the baby’s heartbeat, ask you how you feel and if fine then the check-up is complete. She did not even check the urine. The consultation was complete within 2 minutes.’ (P2, aged 30, prev C/S x 1)

Another participant stated:

‘In my view they were not massaging (palpating the abdomen). The midwife comes and put a something that looks like a microphone, saying they are listening to the baby’s heartbeat, they measure the tummy, check the urine and then collect blood…that’s all they do.’ (P10, aged 28, prev C/S x 1)

#### Use of alternative antenatal care

Some participants preferred ANC services from traditional birth attendants and churches. This was because these alternate ANC providers physically massaged or palpated pregnant women’s abdomens:

‘[*W*]e prefer the old women and the churches … at the clinic it’s not that you get massaged (palpated), it’s just that they have machines which can tell whether the baby is fine and things like scan …’ (P10, aged 28, prev C/S x 1)

#### Experience from previous pregnancies

Some multiparous participants felt that they could detect problems themselves at home:

‘Usually I register at 4 months, but this time around I decided to delay because everything was fine. I also felt that I had experience.’ (P2, aged 30, prev C/S x 1)

#### Antenatal care fatigue

Some participants felt that multiple antenatal visits are tiring:

‘Some people say they don’t want to go to the health facility monthly because it’s tiring. They just want to go when their tummies are big.’ (P7, aged 27, prev C/S x 2)

#### Delay at local clinic

Some participants said that nurses delayed referring them to the high-risk clinic for pregnant women at the hospital. A participant who arrived for the first time in labour noted:

‘I only went for my antenatal visits. They didn’t mention that as the months for delivery drew closer I had to go to LIIMH. They were just giving routine ANC.’ (P5, aged 27, previous C/S x 2)

One of the participants said:

‘I had gone for my usual check-up at the clinic. When I got there they told me that my time is up, that I should have already gone to the hospital so that I could be operated.’ (P9, aged 34, prev C/S x 2)

#### Pregnancy-related factors

Unexpected or unwanted pregnancy, denial and late recognition of pregnancy were reported by some participants to have led to late presentation for ANC services:

A participant who had delivered in the previous year was in denial:

‘I didn’t want to believe that I was pregnant. Since the last child was still young, I didn’t want to believe I was pregnant …’ (P3, aged 34, prev C/S x 1)

Sometimes there is even delay to accept pregnancy by relatives:

‘… Sometimes at home they don’t agree with the pregnancy.’ (P4, aged 30, prev C/S x 1)

## Discussion

This research study explored the reasons why pregnant women with a previous history of C/S deliveries presented after 20 weeks of pregnancy for ANC at LIIMH, Botswana. Participants in this study did not know the necessity of presenting early in pregnancy for ANC. Other studies have also indicated that lack of knowledge of the importance of booking early is an important factor making women present late for ANC.^24,26,27,40,41^ Healthcare workers need to repeatedly engage pregnant patients with previous history of C/S individually, and as groups, so as to explain the importance of reporting at LIIMH not later than 20 weeks. This may help to avoid negative outcomes during delivery in pregnant women with previous history C/S deliveries.

In the absence of effective teaching on ANC by healthcare workers, pregnant women with previous history of C/S may receive wrong ANC education from lay people. Fagbamigbe et al. observed that participants who received information about ANC services from healthcare workers had better knowledge than those who received it from other sources such as friends, relatives and news media.^42^

Participants in this study believed that they were required to present for ANC when the baby was well formed and moving. Other studies have also found misconceptions amongst women (e.g. not being ill, using ANC just to get a card to be admitted to a facility when in labour, going for ANC when the baby is formed) to be a major contributor to late booking for ANC services.^26,43,44^ Continuous engagement with patients and the community on the reasons and processes of ANC may help to change their wrong beliefs and behaviours.

This study showed that participants were not happy with ANC services: they were ‘not massaged (examined)’ satisfactorily, consultation time was short, there were long queues and the clinic opened late. Other studies have also found participant dissatisfaction with ANC services: unsatisfactory examination, long queues,^45,46^ not being taken seriously and lack of information about pregnancy-related issues.^44,47^ The quality of ANC services does not depend solely on the timing and number of ANC visits, and the physical examination but also on the type and quantity of interventions, such as maternal height, urine examination, examining the total blood count, measurement of blood pressure, general physical examination, iron supplementation, administration of folic acid tablets, voluntary counselling and testing for HIV, prevention of maternal transmission of HIV to the infant, administering antimalarial drugs, providing isoniazid prophylaxis against tuberculosis and other interventions.^48,49^ Our study confirms the findings in other studies that showed that ANC interventions though important, were not mentioned as causes of pregnant women’s dissatisfaction with ANC services.

The use of traditional midwives and churches was another reason for late booking of ANC services amongst participants in this study. Seeking ANC services from traditional birth attendants was widely reported amongst pregnant women in Africa.^44,50,51^ The incorporation of traditional birth attendants into the public health sector of Botswana should be considered. Reasons for sourcing ANC from traditional birth attendants in other settings included more experienced and compassionate care by traditional birth attendants,^52^ shortage of professional healthcare providers,^53^ and medical advice and protection from spiritual attacks by churches.^27,54^

Some multiparous participants delayed booking for ANC services because they felt that they were experienced in pregnancy. Other studies have also shown that multiparous women are more likely to delay ANC booking because of the experience from prior pregnancy.^27,41,44^ Four weekly ANC visits until 32 weeks, then fortnightly till term, and then weekly thereafter was tiring for some participants. The challenges of transport to LIIMH situated at the periphery of Maun probably contributed to this feeling. Transport costs and distance from healthcare facilities have been found to contribute to delayed or inadequate use of ANC.^27,41^

Clinic staff sometimes delayed referring patients to LIIMH. Health system issues have been reported as reasons for delay in booking for ANC services in other studies.^46,55^ This study revealed that unexpected and unwanted pregnancies, as well as late realisation of pregnancy, led to delay in booking for ANC services. Unwanted and mistimed pregnancies have been shown to adversely affect ANC initiation.^56,57^ Easier access to and support for contraception, as well as addressing system related factors that dispose pregnant women to suboptimal use of ANC, may help to reduce unplanned and unwanted pregnancies. Such measures may reduce maternal morbidity and mortality rates.

Participants in this study believed that abdominal examination of pregnant women by healthcare workers was just a ‘massage’, as it seems that healthcare workers did not explain the purpose of the obstetric abdominal palpation to patients. We did not find this understanding of obstetric abdominal examination of pregnant women in other studies. Receiving a better ‘massage’ was the main reason participants in this study sought ANC from traditional birth attendants and churches. Perhaps, abdominal palpation of pregnant women needs to be carried out more often during ANC visits. This may increase the perception that ANC visits are worth the effort of travelling to healthcare facilities, and the long waiting times to be attended by a healthcare worker. A minimum of eight ANC visits are recommended, with one visit in the first trimester, two in the second and five in the third trimester.^3^ During these ANC visits, pregnant women should receive recommended nutritional interventions, as well as regular maternal and foetal assessments.

This study confirms reasons for late initiation of ANC services that have been found in other studies. It also shows perhaps for the first time a new understanding (a massage) of the abdominal obstetric examination of pregnant women. The study also highlights health system, pregnancy experience, patient and sociocultural issues that need to be addressed at LIIMH in order to improve appropriate use of ANC by women with a previous history of C/S delivery.

## Conclusion

Pregnant women with a previous history of C/S delivery presented late for ANC because of lack of information from healthcare providers, dissatisfaction with ANC, experience gained from multiparity, use of alternative ANC providers, unwanted pregnancy, unexpected pregnancy and late recognition of pregnancy. Participants favoured obstetric abdominal examination of a pregnant woman, and believed that it was a massage and did not understand it as diagnostic. These findings suggest that the problem of late registration for ANC services by pregnant women with a previous history of C/S should be addressed holistically at individual, family, community and health-system levels. Healthcare workers also need to provide pregnant women with correct information on ANC and about the exact date they need to be transferred or referred to LIIMH for appropriate care.

### Evidence from this study suggests the following recommendations

- Healthcare workers should be sensitised to giving correct information to pregnant women with a previous history of C/S delivery, and they should refer pregnant women with a previous history of C/S to LIIMH before 20 weeks gestation.
- Antenatal care delivery in clinics and primary hospitals should be user friendly, and efforts should be made for all stakeholders to work harmoniously with pregnant women.
- The importance of having the required number of ANC visits and starting ANC visits in the first trimester should be discussed with women in village fora and during ANC visits.
- The Botswana government should explore the incorporation of traditional birth attendants into the public Botswana healthcare sector for communication and support.

## Appendix 1

**TABLE 1-A1:** Interview guide.

**Demographic data**				
Age:	< 20 □	20–24 □	25–29 □	30+ □
Marital status:	Single □	Married □	Widowed □	Divorced □
Education level:	None □	Primary □	Secondary □	Tertiary □
Residence				
Previous obstetric Hx				
Parity:	1–2 □	3–4 □	5+ □	
Number of previous C/Ss:	1 □	2 □	3+ □	
Date of last pregnancy (years):	1 □	2 □	2+ □	
**Interview guide** Why did you register after 20 weeks of pregnancy at LIIMH? Probe: Please tell me about other reasons why you registered after 20 weeks. In your view why do some pregnant women with previous caesarean section (C/S) register after 20 weeks of pregnancy for antenatal care (ANC)? Probe: Some pregnant women with previous C/S register for ANC after 20 weeks of pregnancy. Please tell me possible reasons why these patients register for ANC after 20 weeks. Probe: Please give me other reasons why you think some pregnant women with previous C/S register after 20 weeks for ANC. What do people say are the reasons why some pregnant women with previous C/S register for ANC after 20 weeks? Probe: Please tell me other reasons that people say make women register for ANC after 20 weeks. What do you think can be done to help pregnant women with previous C/S to register for ANC before 20 weeks of pregnancy? Probe: Please tell me other things that can be done to help women with previous C/S to register before 20 weeks of pregnancy. What do people say should be done to help pregnant women with previous C/S to register before 20 weeks for ANC? Probe: What are other things that people say should be done to help pregnant women with previous c/s to register for ANC before 20 weeks? Probe: Please tell me what you think nurses can do to help pregnant women with previous C/S to register for ANC before 20 weeks? Probe: What else can nurses do to help pregnant women with previous C/S to register for ANC before 20 weeks? Probe: Please tell me what you think doctors can do to help pregnant women with previous c/s to register for ANC before 20 weeks. Probe: What else can doctors do to help pregnant women with previous C/S to register for ANC before 20 weeks? What do you think should happen in hospitals and clinics to help pregnant women with previous c/s to register for ANC before 20 weeks of pregnancy?				

Hx, history; C/S, caesarean section; ANC, antenatal care; LIIMH, Letsholathebe II Memorial Hospital.

## References

[1] Government of Botswana and the UNDP. MDG acceleration compact. Reduce maternal mortality in Botswana: Three years to go. In: Ministry of Health of Botswana, editor. Gaborone: Government of Botswana and UNDP, 2013; p. 19–23.

[2] Ray S, Madzimbamuto FD, Ramagola-Masire D, et al. Review of causes of maternal deaths in Botswana in 2010. S Afr Med J. 2013;103(8):537–542. 10.7196/SAMJ.6723 23885735

[3] World Health Organisation (WHO). WHO recommendations on antenatal care for a positive pregnancy experience: Summary. Geneva: WHO; 2018. 28079998

[4] Cantwell R, Clutton-Brock T, Cooper G, et al. Saving mothers’ lives: Reviewing maternal deaths to make motherhood safer: 2006–2008. The eighth report of the confidential enquiries into maternal deaths in the United Kingdom. BJOG: Int J Obstet Gynaecol. 2011;118(Suppl 1):1–203. 10.1111/j.1471-0528.2010.02847.x 21356004

[5] WHO, UNICEF, UNFPA, World Bank Group, Division UNP. Trends in martenal mortality 2000–2017. Geneva: World Health Organisation; 2019.

[6] Shennan AH, Green M, Chappell LC. Maternal deaths in the UK: Pre-eclampsia deaths are avoidable. Lancet. 2017;389(10069):582–584. 10.1016/S0140-6736(17)30184-8 28195043

[7] MBRRACE-UK. Saving lives, improving mothers’ care – Lessons learned to inform maternity care from the UK and Ireland confidential enquiries into maternal deaths and morbidity 2013–2015. Oxford: National Perinatal Epidemiology Unit, University of Oxford; 2017.

[8] Mogobe KD, Tshiamo W, Bowelo M. Monitoring maternity mortality in Botswana. Reprod Health Matters. 2007;15(30):163–171. 10.1016/S0968-8080(07)30330-3 17938081

[9] Statistics Botswana. Botswana – Maternal mortality ratio 2017. Gaborone: Statistics Botswana; 2019.

[10] Sinvula M, Insua M. Botswana maternal mortality reduction initiative: Final report Bethseda, MD: University Research Co., LLC (URC); USAID, 2015.

[11] Madzimbamuto F, Ray SC, Mogobe KD, et al. A root-cause analysis of maternal deaths in Botswana: Towards developing a culture of patient safety and quality improvement. BMC Pregnancy Childbirth. 2014;14:231. 10.1186/1471-2393-14-231 25030702 PMC4223720

[12] Moller A-B, Petzold M, Chou D, Say L. Early antenatal care visit: A systematic analysis of regional and global levels and trends of coverage from 1990 to 2013. Lancet Glob Health. 2017;5(10):E977–E983. 10.1016/S2214-109X(17)30325-X 28911763 PMC5603717

[13] De Graft-Johnson J, Kerber K, Tinker A, et al. The maternal, newborn, and child health continuum of care. In: Lord D, Wake R, Elder L, Grear K, Antayhua A, editors. Opportunities for Africa’s Newborns. Cape Town: Mills Litho, 2012; p. 250.

[14] Lincetto O, Mothebesoane-Anoh S, Gomez P, Munjanja S. Antenatal care. In: Lord D, Wake R, Elder L, Grear K, A. A, editors. Opportunities for Africa’s Newborns. Cape Town: Mills Litho, 2012; p. 250.

[15] Aduloju OP, Akintayo AA, Ade-Ojo IP, Awoleke JO, Aduloju T, Ogundare OR. Gestational age at initiation of antenatal care in a tertiary hospital, Southwestern Nigeria. Niger J Clin Pract. 2016;19(6):772–777. 10.4103/1119-3077.181398 27811450

[16] Aung TZ, Oo WM, Khaing W, Lwin N, Dar HT. Late initiation of antenatal care and its determinants: A hospital based cross-sectional study. Int J Community Med Public Health. 2016;3(4):900–905. 10.18203/2394-6040.ijcmph20160926

[17] Sibeko S, Moodely J. Healthcare attendance patterns by pregnant women in Durban, South Africa. S Afr Fam Pract. 2006;48(10):17–17e. 10.1080/20786204.2006.10873478

[18] Sibiya MN, Ngxongo TSP, Reddy P, et al. Timing of first antenatal care attendance and associated factors among pregnant women in an obstetric health facility in eThekweni district, KwaZulu-Natal, South Africa. J Phys Act Health. 2018;24(2):181–192.

[19] Geta MB, Yallew WW. Early initiation of antenatal care and factors associated with early antenatal care initiation at health facilities in Southern Ethiopia. Adv Public Health. 2017;2017:1624245. 10.1155/2017/1624245

[20] Esscher A, Binder-Finnema P, Bødker B, Högberg U, Mulic-Lutvica A, Essén B. Suboptimal care and maternal mortality among foreign-born women in Sweden: Maternal death audit with application of the ‘migration three delays’ model. BMC Pregnancy Childbirth. 2014;14:141. 10.1186/1471-2393-14-141 24725307 PMC3998732

[21] Kuhnt J, Vollmwer S. Antenatal care services and its implications for vital and health outcomes of children: Evidence from 193 surveys in 69 low-income and middleincome countries. BMJ Open. 2017;7:e017122. 10.1136/bmjopen-2017-017122 PMC5695442 29146636

[22] Mathe M. Sociodemographic factors affecting utilization of ante natal care services in Botswana. Int J Acad Res Bus Soc Sci. 2017;7(9):477–520. 10.6007/IJARBSS/v7-i9/3343

[23] WHO. Ante natal care coverage: Data by country. Geneva: Global Health Depository data repository; 2020.

[24] Jones GL, Haddrill R, Mitchell C, Anumba DA. Why do women attend late for antenatal booking? A qualitative interview study exploring the perspectives of service users and stakeholders. J Epidemiol Community Health. 2011;65(Suppl II):A4. 10.1136/jech.2011.143586.9

[25] Okedo-Alex IN, Akamike IC, Ezeanosike OB, Uneke CJ. Determinants of antenatal care utilisation in sub-Saharan Africa: A systematic review. BMJ Open. 2019;9:e031890. 10.1136/bmjopen-2019-031890 PMC6797296 31594900

[26] Ebeigbe PN, Igberase GO. Reasons given by pregnant women for late initiation of antenatal care in the Niger Delta, Nigeria. Ghana Med J. 2010;44(2):47–51. 10.4314/gmj.v44i2.68883 21327003 PMC2994152

[27] Jinga N, Mongwenyana C, Moolla A, Malete G, Onoya D. Reasons for late presentation for antenatal care, healthcare providers’ perspective. BMC Health Serv Res. 2019;19:1016. 10.1186/s12913-019-4855-x 31888616 PMC6937646

[28] Gebrekidan K, Worku A. Factors associated with late ANC initiation among pregnant women in select public health centers of Addis Ababa, Ethiopia: Unmatched case–control study design. Pragmatic Observational Res. 2017;8:223–230. 10.2147/POR.S140733 PMC5667792 29138615

[29] Paudel YR, Jha T, Mehata S. Timing of first antenatal care (ANC) and inequalities in early initiation of ANC in Nepal. Front Public Health. 2017;5:242. 10.3389/fpubh.2017.00242 28955707 PMC5600995

[30] Hassan A. Trial of scar and vaginal birth after caesarean section. J Ayub Med Coll Abbottabad. 2005;17(1):57–61. 15929530

[31] Muppala H, Najia SK, Clarke FR. Current evidence in the management of previous caesarean section: Clinical review. Eur Clin Obstet Gynaecol. 2007;3:67–80. 10.1007/s11296-007-0065-x

[32] Fitzpatrick KE, Kurinczuk JJ, Bhattacharya S, Quigley MA. Planned mode of delivery after previous cesarean section and short-term maternal and perinatal outcomes: A population-based record linkage cohort study in Scotland. PLoS Med. 2019;16(9):e1002913. 10.1371/journal.pmed.1002913 31550245 PMC6759152

[33] Denham SH, Humphrey T, De Labrusse C, Dougall N. Mode of birth after caesarean section: Individual prediction scores using Scottish population data. BMC Pregnancy Childbirth. 2019;19:84. 10.1186/s12884-019-2226-6 30819140 PMC6396527

[34] Sobhy S, Arroyo-Manzano D, Murugesu N, et al. Maternal and preinatal mortality and complications associated with caesarean section in low-income and middle-income countries: A systematic review and meta-analysis. Lancet. 2019;393(10184):1973–1982. 10.1016/S0140-6736(18)32386-9 30929893

[35] Gurung P, Malla S, Lama S, Malla A, Singh A. Caesarean section during second stage of labor in a Tertiary Centre. J Nepal Health Res Counc. 2017;15(2):178–181. 10.3126/jnhrc.v15i2.18210 29016591

[36] Gupta JK, Smith GCS, Chodankar RR. Birth after previous caesarean birth green-top guideline no. 45, October 2015. London: Royal College of Obstetricians and Gynaecologists; 2015.

[37] Botswana S. Botswana in Figures. Gaborone: Statistics Botswana, 2016; p. 40–49.

[38] Linneberg MS, Korsgaard S. Coding qualitative data: A synthesis guiding the novice. Qual Res J. 2019;19(3):259–270. 10.1108/QRJ-12-2018-0012

[39] Mabuza LH, Govender I, Ogunbanjo GA, Mash B. African primary care research: Qualitative data analysis and writing results. Afr J Prim Health Care Fam Med. 2014;6(1):a640. 10.4102/phcfm.v6i1.640 PMC4502868 26245437

[40] Gudayu TW, Woldeyohannes SM, Abdo AA. Timing and factors associated with first antenatal care booking among pregnant mothers in Gondar Town, North West Ethiopia. BMC Pregnancy Childbirth. 2014;14:287. 10.1186/1471-2393-14-287 25154737 PMC4152591

[41] Mgata S, Maluka SO. Factors for late initiation of antenatal care in Dar es Salaam, Tanzania: A qualitative study. BMC Pregnancy Childbirth. 2019;19:415. 10.1186/s12884-019-2576-0 31718586 PMC6849280

[42] Fagbamigbe AF, Akanbiemu FA, Adebowale AS, Olumide AM, Korter G. Practice, knowledge and perceptions of antenatal care services among pregnant women and nursing mothers in Southwest Nigeria. Int J Matern Child Health. 2013;1(1):7–16. 10.12966/ijmch.05.02.2013

[43] Ifenne DI, Utoo BT. Gestational age at booking for antenatal care in a tertiary health facility in north-central, Nigeria. Niger Med J. 2012;53(4):236–239. 10.4103/0300-1652.107602 23661885 PMC3640246

[44] Kisuule I, Kaye DK, Najjuka F, et al. Timing and reasons for coming late for the first antenatal care visit by pregnant women at Mulago hospital, Kampala Uganda. BMC Pregnancy Childbirth. 2013;13:121. 10.1186/1471-2393-13-121 23706142 PMC3665546

[45] Chemir F, Alemseged F, Workneh D. Satisfaction with focused antenatal care service and associated factors among pregnant women attending focused antenatal care at health centers in Jimma town, Jimma zone, South West Ethiopia: A facility based cross-sectional study triangulated with qualitative study. BMC Res Notes. 2014;7:164. 10.1186/1756-0500-7-164 24646407 PMC3994781

[46] Abrahams N, Jewkes R, Mvo Z. Health care – Seeking practices of pregnant women and the role of the midwife in Cape Town, South Africa. J Midwifery Womens Health. 2001;46(4):240–247. 10.1016/S1526-9523(01)00138-6 11603639

[47] Hildingsson I, Haines H, Cross M, Pallant JF, Rubertsson C. Women’s satisfaction with antenatal care: Comparing women in Sweden and Australia. Women Birth. 2013;26(1):e9–e14. 10.1016/j.wombi.2012.06.002 22795867

[48] Kyei NNA, Chansa C, Gabrysch S. Quality of antenatal care in Zambia: A national assessment. BMC Pregnancy Childbirth. 2012;12:151. 10.1186/1471-2393-12-151 23237601 PMC3536568

[49] Katemba BM, Bwembya P, Hamoonga TE, Chola M, Jacobs C. Demand side factors associated with quality antenatal care services: A case study of Lusaka district, Zambia. Front Public Health. 2018;6:285. 10.3389/fpubh.2018.00285 30356734 PMC6189466

[50] Selepe HL, Thomas DJ. The beliefs and practices of traditional birth attendants in the Manxili area of KwaZulu, South Africa: A qualitative study. J Transcult Nurs. 2000;11(2):96–101. 10.1177/104365960001100203 11982050

[51] Van Eijk AM, Bles HM, Odhiambo F, et al. Use of antenatal services and delivery care among women in rural western Kenya: A community based survey. Reprod Health. 2006;3(2). 10.1186/1742-4755-3-2 PMC1459114 16597344

[52] Oyerinde K, Harding Y, Amara P, et al. A Qualitative evaluation of the choice of traditional birth attendants for maternity care in 2008 Sierra Leone: Implications for universal skilled attendance at delivery. Matern Child Health J. 2013;17(5):862–868. 10.1007/s10995-012-1061-4 22736032

[53] Regulation Gazette 30165. Government notice R172. South Africa: Government Printers, Government notice R172. 2007.

[54] Etuk SJ, Itam IH, Asuquo EE. Role of the spiritual churches in antenatal clinic default in Calabar, Nigeria. East Afr Med J. 1999;76(11):639–643. 10734525

[55] Mrisho M, Obrist B, Schellenberg JA, et al. The use of antenatal and postnatal care: Perspectives and experiences of women and health care providers in rural southern Tanzania. BMC Pregnancy Childbirth. 2009;9(10). 10.1186/1471-2393-9-10 PMC2664785 19261181

[56] Amon Exavery A, Kanté AM, Hingora A, Mbaruku G, Pemba S, Phillips JF. How mistimed and unwanted pregnancies affect timing of antenatal care initiation in three districts in Tanzania. BMC Pregnancy Childbirth. 2013;13:35. 10.1186/1471-2393-13-35 23388110 PMC3574825

[57] Dibaba Y, Fantahun M, Hindin MJ. The effects of pregnancy intention on the use of antenatal care services: Systematic review and meta-analysis. Reprod Health. 2013;10:50. 10.1186/1742-4755-10-50 24034506 PMC3848573

